# GRP78 autoantibodies initiate the breakdown of the blood-brain barrier in neuromyelitis optica

**DOI:** 10.18632/oncotarget.22589

**Published:** 2017-11-21

**Authors:** Fumitaka Shimizu, Richard M. Ransohoff

**Affiliations:** Fumitaka Shimizu: Department of Neurology and Clinical Neuroscience, Yamaguchi University Graduate School of Medicine, Minamikogushi, Ube, Yamaguchi, Japan

**Keywords:** blood-brain barrier, neuromyelitis optica, glucose-regulated protein 78, anti-endothelial cell antibodies

Antibody-based therapy offers vast potential in the treatment of several neurodegenerative diseases. Many drug companies are now engineering antibodies targeting β-amyloid protein for Alzheimer disease and α-synuclein protein for Parkinson disease. However, the development of antibody-based therapies against the central nervous system (CNS) diseases has lagged, partly due to the poor blood-brain barrier (BBB) permeability of large molecules [1]. In a recent study published in Science Translational Medicine [2], we searched for targets of anti-endothelial cell antibodies that can modulate BBB permeability in patients with neuromyelitis optica (NMO).

NMO is a disabling autoimmune CNS disease. The main clinical manifestations of NMO are recurrent optic neuritis and longitudinal transverse myelitis [3]; however, widespread lesions are frequently observed throughout the brain and brain stem. The discovery of serum autoantibodies for the water channel aquaporin-4 (AQP4) expressed on astrocyte endfeet (known as NMO-IgG or AQP4-IgG) has highlighted NMO as a distinct clinical entity from multiple sclerosis [4]. The perivascular binding pattern of NMO-IgG on rodent brain tissue sections was consistent with reactivity against AQP4, which is predominantly found on astrocyte foot processes forming the glia limitans of the BBB. The presence of serum AQP4-IgG has facilitated the clinical diagnosis and early treatment of NMO patients worldwide [4]. Antibody-depleting therapy and B-cell targeting therapy for the removal of AQP4-IgG are effective for reducing the disease activity in NMO patients. Several lines of experimental evidence support that the binding of AQP4-IgG to AQP4 on astrocytes initiates complement- and antibody-dependent cellular cytotoxicity in target astrocytes. Thus, NMO is considered to be the first CNS autoimmune disease for which a specific tissue target molecule has been identified.

AQP4-IgG is mostly produced by peripheral plasmablasts rather than intrathecal plasmablasts, which suggests that CSF AQP4-IgG is derived from serum. However, the presence of serum AQP-IgG alone is not sufficient to cause NMO disease [5]. Although the peripheral injection of AQP4-IgG did not induce NMO-like histopathology when injected into naive animals, it caused disease in experimental animals with pre-existing T-cell mediated CNS inflammation. AQP4-IgG was detectable in the sera of some NMO patients for many years before the onset of the disease, which suggests that AQP4-IgG can persist in the circulation without causing any evident disease. Thus, the BBB breakdown that allows for the subsequent penetration of AQP4-IgG into the brain seems a prerequisite for the development of NMO. Clinical observations demonstrating the Gd-enhanced lesions on MRI, increased albumin leakage in the cerebrospinal fluid (CSF) in the acute phase of NMO, and diffuse perivascular lesions along with pathological evidence of the disruption of the BBB would support the significance of BBB disruption in the pathogenesis of NMO.

How circulating AQP4-IgG can access the AQP4 on the astrocyte endfeet behind the BBB in NMO is poorly understood. One possible route is through circumventricular organs such as the area postrema, where the endothelial cells lack tight junctions and the AQP4 expression is enriched [6], as indicated by the observation that NMO lesions are frequently observed in the hypothalamus and the periaqueductal brainstem surrounding the circumventricular system. After AQP4-IgG enters the CSF space, it can freely access the target astrocytes and cause BBB dysfunction via intrathecal inflammation.

**Figure 1:**
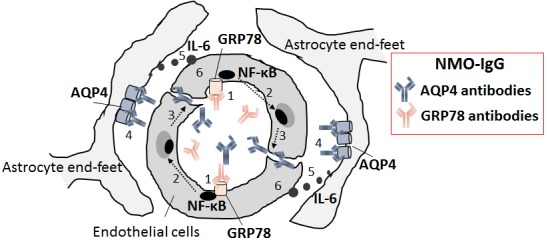
The effect of GRP78 antibodies on the pathogenesis of NMO 1. The binding of GRP78 autoantibodies to cell surface GRP78. 2. The induction of NF-κB nuclear translocation. 3. The enhancement of macromolecular permeability and the penetration of AQP4 antibodies into the brain. 4. The binding of AQP4 antibodies to AQP4 on astrocytes. 5. The production of IL-6 by astrocytes. 6. The activation of IL-6 signaling in endothelial cells and the further induction of blood-brain barrier breakdown.

The other possible route is via direct BBB transit. However, serum AQP4-IgG cannot open the BBB itself, as the BBB-endothelial cells do not express the AQP4 protein. We recently identified a distinct endothelial cell targeted-autoantibody that enhances the BBB transit of AQP4-IgG in NMO [2]. First, we identified two AQP4-nonreactive monoclonal rAbs prepared from CSF plasmablasts in NMO patients, which can bind and activate the human brain microvascular endothelial cells (BMECs) via NF-κB p65 signaling *in vitro*. Next, we identified glucose-regulated protein 78 (GRP78) as the target antigen of this NMO-rAb. Last, we confirmed that the peripheral administration of a GRP78-specific NMO-rAb resulted in increased BBB leakage *in vivo*. Our results suggest that GRP78 autoantibodies directly contribute to increased BBB leakage and NMO attacks in at least some NMO patients (Figure 1). Our recent findings suggest that the binding of AQP4-IgG to AQP4 can induce inflammation via the production of IL-6 by astrocytes and that subsequent IL-6 signaling to BMECs gave rise to the further breakdown of the BBB [7] (Figure 1).

Endothelial cell autoantibodies have been commonly described in systemic lupus erythematosus (SLE) for over a decade; however, their target antigen has not been identified. NMO patients often have coexisting SLE and AQP4 autoantibodies have been found in SLE patients with neurological signs consistent with NMO. We also demonstrated that IgG from SLE patients lead to the activation of endothelial cells in the BBB, suggesting that GRP78 autoantibodies, as a target of anti-endothelial cell antibodies, may be detectable in some SLE patients. GRP78 autoantibodies have also been detected in the sera from patients with cancer and rheumatoid arthritis, and have been reported as a potential biomarker that can be used to make an early diagnosis [8]. We hypothesize that GRP78 autoantibodies only have a pathogenic effect on CNS dysfunction when accompanied by a CNS-specific autoantibody, such as AQP4 autoantibodies.

In addition to providing a clinical biomarker that can be used to predict an NMO attack, the discovery of GRP78 autoantibodies from NMO patients also provides a candidate target for promoting the CNS transit of therapeutic antibodies for many CNS diseases. Further research is needed to establish the GRP78-based delivery of therapeutic antibodies in the CNS.
